# Breast cancer patients with isolated bone metastases and oligometastatic bone disease show different survival outcomes

**DOI:** 10.1038/s41598-021-99726-7

**Published:** 2021-10-11

**Authors:** Baha Zengel, Mustafa Kilic, Funda Tasli, Cenk Simsek, Murat Karatas, Ozlem Ozdemir, Demet Cavdar, Raika Durusoy, Kadir Koray Bas, Adam Uslu

**Affiliations:** 1 Department of General Surgery, Izmir Bozyaka Health Practice and Research Center, University of Health Sciences Turkey, Izmir, Turkey; 2 Department of Pathology, Izmir Bozyaka Health Practice and Research Center, University of Health Sciences Turkey, Izmir, Turkey; 3 Department of Medical Oncology, Izmir Bozyaka Health Practice and Research Center, University of Health Sciences Turkey, Izmir, Turkey; 4 Department of Public Health, Medical Faculty, Ege University, Izmir, Turkey

**Keywords:** Medical research, Oncology, Risk factors

## Abstract

In this study, we planned to investigate the clinical course of patients with breast cancer with oligometastatic bone disease (OMBD). The patients were grouped according to the characteristics and the sites of metastases. Group I included 928 patients without metastasis. Group II, the OMBD group, included 68 patients. Group III, the widespread metastasis group, comprised 185 patients with multiple bone metastases and/or solid organ metastases. The mean overall survival of the groups was 16.7 ± 0.3 years in group 1, and 7.8 ± 0.8 and 5.9 ± 0.4 years in groups 2 and 3, respectively (*p* < 0.001 for the comparison of all three groups together; *p* < 0.001 for group 1 vs. 2 and 3) and (*p* = 0.037 for group 2 vs. group 3). In the subgroup survival analysis of patients in group 2 (OMBD), the mean and median survival was 5.5 ± 0.8 and 4.0 ± 0.8 years vs. 9.2 ± 0.98 and 9.0 ± 1.05 years in patients with more than one bone metastasis and single bone metastasis, respectively (*p* = 0.019). OMBD seems to be a different disease than breast cancer with isolated bone metastases. The high risk of developing OMBD, especially following locoregional recurrence, increases the importance of locoregional therapy in large T and N stage tumors.

## Introduction

Breast carcinoma is a tumor with osteotropic potential and the most common cause of carcinoma-related deaths in women^1,2^. Nearly 70% of the patients who die of breast cancer have evidence of metastatic bone disease at autopsy^3^. Models for predicting the effect of variables on breast cancer mortality have estimated a median of 19% reduction attributable to adjuvant therapy alone^4^. In a recent update study, the addition of targeted therapies to a chemotherapeutic agent has improved median overall survival (OS) up to 56.5 months in patients with HER2-positive metastatic breast cancer^5^. The survival outcomes of patients with stage IV breast cancer vary according to metastatic site and those with bone metastasis have the best survival^6^. In this context, oligometastatic breast cancer generally refers to a special group of patients with fewer than five metastatic deposits in a single organ and is considering potentially curable stage IV disease^7^. However, the definition of oligometastatic bone disease (OMBD) in the literature varies according to the number and location of metastasis^8^. It is still uncertain whether OMBD corresponds to an intermediate stage between localized and widespread disease or a genetically unique entity, rather than a transition point from a primary tumor to metastasis^9^.

**Figure 1:**
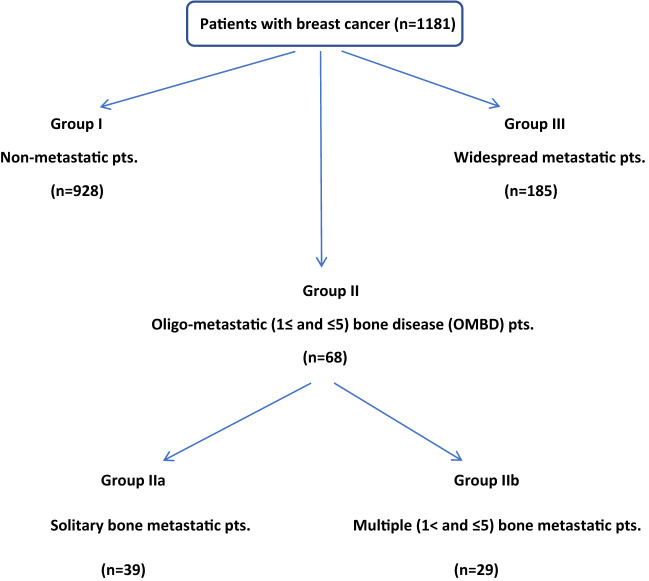
Patient groups in this study. GROUP I: non-metastatic group. GROUP II: oligometastatic bone disease (OMBD) = oligo-bone metastasis group = single bone metastases (SBM). (IIa) Patients with solitary bone metastasis = Only one bone met. (IIb) Patients with oligo bone metastasis = More than one but fewer than five bone metastases. GROUP III: Widespread metastatic disease = solid organ metastasis and/or multiple (more than five) bone metastasis groups.

In this study, with inspiration from the current literature and our previous publication about clinicopathologic features of single bone metastasis (SBM) in breast cancer^10^, we planned to investigate the clinical course of patients with breast cancer with OMBD. We evaluated the demographic features of patients, histopathologic features with intrinsic subtypes of tumors and treatment-related factors on “survival outcomes” among a non-metastatic group (group I), an OMBD group (group II), and a widespread metastatic group that included patients with solid organ metastases with or without bone metastasis (group III) (Fig. 1). Also, we aimed to determine the common characteristics of the patients with solitary (only one) and oligo (more than 1 but fewer than or equal to 5) bone metastasis in the OMBD group by evaluating them in terms of clinico-pathologic factors and survival outcomes. For this purpose, a sub-group analysis was conducted to compare two strata of the OMBD group (group II), comparing patients with solitary bone metastasis (group IIa) and patients with OMBD (group IIb).

## Materials and methods

This retrospective cohort study was performed at Izmir Bozyaka Health Practice and Research Center, University of Health Sciences, Turkey, and was prepared for publication following the approval of the ethics committee on May 6th, 2020. The study included consecutive patients with breast cancer who underwent surgery between 2000 and 2020 in the Department of General Surgery. Those, who were aged 23–92 years, had completed adjuvant therapy, had regular database and follow-ups, and were followed up for at least 6 months were included.

There were a total of 1181 patients (1175 women, 6 men) in our series. The patients were grouped according to the characteristics and the sites of metastases. Group I included 928 patients without metastasis. Group II, the OMBD group, constituted 68 patients. Group III, the widespread metastasis group, comprised 185 patients with multiple (more than six) bone metastases and/or solid organ metastases.

Between 2000 and 2015, we performed whole-body bone scintigraphy (B-scan) and/or magnetic resonance imaging (MRI) to determine bone metastases. After 2015, bone metastases were detected using B-scan and/or computed tomography and confirmed with 18-fluorodeoxyglucose (FDG) whole-body positron emission tomography (PET)/CT in all cases. Also, these patients were evaluated using thoraco-abdominal CT and PET/CT to detect any concomitant solid organ metastasis.

During the follow-up period, routine bone scintigraphy was performed in patients with bony symptoms and elevated tumor markers (CEA, CA15-3) and those with high-risk tumors including TNM Stage III tumors and mixed-type (invasive ductal carcinoma + invasive lobular carcinoma) histology. In addition, women with a history of breast cancer may be at increased risk of osteoporosis as a result of prior cancer treatment. For this reason, bone scanning with dual-energy X-ray absorptiometry (DEXA) was also performed on the following patients: Women over age 65 years or aged 60 to 64 years in the presence of any of the following: (1) a family history of osteoporosis; (2) body weight < 70 kg; (3) a history of a nontraumatic fracture or other risk factors for osteoporosis (e.g. smoking, sedentary lifestyle, alcohol use) or postmenopausal women taking an aromatase inhibitor (AI) or premenopausal women who develop treatment-related premature menopause. Bisphosphonate therapy was initiated in women with two or more risk factors and pre-existing osteopenia or osteoporosis. Patients in the risk group received adjuvant bisphosphonate therapy with zoledronic acid or oral bisphosphonate for 3–5 years.

Besides radiologic diagnoses, histopathologic diagnoses of bone metastases were available in only 5 of 68 cases. Of these, two patients underwent bone biopsy, and three underwent total excision of metastatic bone fragments of the pathologic fractures.

The groups were compared in terms of demography, treatments received, histopathologic features, and TNM stages of the American Joint Committee on Cancer (AJCC). In demographic factors, body mass index (BMI), smoking, family history, menopausal status, co-morbidities, and hormone use were investigated. The history of hormone use described oral contraceptive (OC) drugs for pre-menopausal and estrogen-progesterone combinations in postmenopausal patients. Hormone replacement therapy (HRT) refers to regular hormone therapy taken at any time, up to the diagnosis of breast cancer. Co-morbidity in patients refers to hypertensive atherosclerotic heart disease, chronic obstructive pulmonary disease, congestive heart failure, cerebrovascular disease, and autoimmune diseases. Treatment factors included the type of breast surgery [mastectomy (M), breast-conserving surgery (BCS)], axillary intervention [axillary lymph node dissection (ALND), sentinel lymph node biopsy (SLNB)], neoadjuvant- CT (NACT), radiotherapy (RT) and hormone therapy (HT). Histopathologic features and staging explain tumor location, histologic and nuclear grade, mitotic activity, perinodal involvement, receptor status, cerb2, E-cadherin, p53, Ki67, lymph vessel invasion, molecular classification [luminal A-B, triple-negative, human epidermal growth factor receptor 2 (HER2)(+)], TNM staging, and local recurrence.

Molecular subtypes of breast cancer are defined as @@follows:

- Luminal A: Hormone-receptor positive (HR+  estrogen-receptor and/or progesterone-receptor (PR) positive), HER2-negative, low Ki-67 levels, and nuclear grade (Grade I).
- Luminal B: HR+ and HER2-positive or HR+ with high Ki-67 levels but HER2-negative. Nuclear grade is moderate or high (Grade II–III).
- Triple-negative/basal-like: HR-negative and HER2-negative. Nuclear grade is moderate or high (Grade II–III).
- HER2-enriched: HR-negative and HER2-positive. Nuclear grade is high (Grade-III).

In this study, the TNM classification of the AJCC was used for staging. Estrogen receptor (ER), PR, HER2, an the Ki-67 proliferation index were evaluated using immunohistochemical staining. Positive nuclear reaction (≥ 1%) for ER and PR and 3 + immunohistochemical staining for HER-2 were recorded as positive. Immunohistochemical staining intensity of 2 + for HER-2 was checked using fluorescence in situ hybridization (FISH) in all patients. The positive cut-off value for Ki67 in immunohistochemical staining was determined as 14%.

In our center, HER-2 analysis was started in 2011 and continued uninterruptedly in all patients thereafter. For treatment schemas, all patients who had Luminal B, triple-negative, and HER2 disease received chemotherapeutics containing anthracycline and taxane, if not contraindicated by their ECHO results. Patients with luminal A breast cancer also received chemotherapy until the mid-2000s, even if they had OMBD, but they then received endocrine-only treatment, together with bisphosphonates. In addition, all patients with HER-2 positive disease received trastuzumab treatment by the time of their diagnosis. Adjuvant bisphosphonates are not considered as a standard of care in Turkey, thus only patients with a high risk of osteoporosis received them every 6 months, and for any type of bone metastasis, all patients received bisphosphonates as a part of their therapy.

### Ethics statement

Ethics Committee Approval: Authors declared that the research was conducted according to the principles of the World Medical Association Declaration of Helsinki “Ethical Principles for Medical Research Involving Human Subjects” (amended in October 2013). The ethical approval for this study was obtained from University of Health Sciences Turkey, Izmir Bozyaka Health Practice and Research Center, Clinical Research Ethics Committee (Date: 12.05.2020 Decision no: 07).

### Informed consent

Written informed consent was obtained from patients who participated in this study.

## Statistics

In univariate analyses, the patients in the three groups were compared using the Chi-square test for categorical variables and Student’s t-test for continuous variables. Two separate logistic regression models were developed using the backward likelihood ratio method with variables found significant in univariate analyses, one exploring independent factors associated with isolated and/or oligo-bone metastasis (group II), and the other predicting independent risk factors of multiple bone metastases and/or solid organ metastases (group III), both compared with the non-metastatic group (group I). Odds ratios (OR) and 95% confidence intervals (CI) were calculated for each possible determinant adjusted for other variables in the model. Survival times and survival curves were calculated and plotted using Kaplan–Meier analysis. Also, patients with single bone metastasis (SBM) were compared with patients with more than one bone metastasis in terms of survival outcomes using the Chi-square, Student t, and Mann–Whitney U tests. P-values of less than 0.05 were considered significant.

## Results

There was no significant difference in the history and demographic parameters except for tumor markers (Table 1). CEA and CA 15-3 values were statistically significantly different between the groups.

**Table 1:** Demographics and history.

Demographics and history		Group 1	Group 2	Group 3	*p*
Age	Median (range)	54 (23–92)	51.5 (28–82)	51 (24–84)	0.086
**BMI**					
Underweight	n (%)	5 (0.8)	1 (2.3)	3 (2.9)	0.476
Normal		155 (25.3)	10 (22.7)	23 (22.3)	
Overweight		226 (36.9)	17 (38.6)	44 (42.7)	
Obese		227 (37.0)	16 (36.4)	33 (32.0)	
BMI	Median (range)	28.3	27.8	27.8	0.737
		(14.9–51.3)	(18.3–44.3)	(16.5–48.3)	
**Smoking**					
No	n (%)	471 (65.1)	38 (76.0)	82 (66.1)	0.287
Yes		253 (34.9)	12 (24.0)	42 (33.9)	
**Hormone use**					
No	n %	407 (52.9)	30 (60.0)	80 (63.5)	0.064
OC or HRT		164 (21.3)	15 (30.0)	24 (19.0)	
OC		145 (18.9)	4 (8.0)	17 (13.5)	
HRT		31 (4.0)	0 (0)	5 (4.0)	
OC + HRT		22 (2.9)	1 (2.0)	0 (0)	
**Diabetes**					
No	n %	684 (84.4)	51 (89.5)	116 (86.6)	0.509
Yes		126 (15.6)	6 (10.5)	18 (13.4)	
**Comorbid disease**					
No	n %	221 (47.5)	15 (60.0)	30 (48.4)	0.477
Yes		244 (52.5)	10 (40.0)	32 (51.6)	
**Family history**					
No	n %	607 (77.1)	45 (83.3)	98 (76.0)	0.531
Yes		180 (22.9)	9 (16.7)	31 (24.0)	
**Menopausal status**					
Premenopausal	n %	329 (36.3)	27 (40.3)	72 (41.4)	0.161
Postmenopausal		573 (63.3)	40 (59.7)	99 (56.9)	
Male		3 (0.4)	0 (0)	3 (1.7)	
CEA	Median (range)	1.7	2	2.2	< 0.001
		(0.2–56.2)	(0.4–26.1)	(0.2–312.1)	
CA15-3	Median (range)	15.1	18.2	20.1	< 0.001
		(0.5–333.7)	(4–127.1)	(5.8–698.5)	

*BMI* body mass index, *OC* oral contraceptives, *HRT* hormone replacement therapy.

The surgical treatment performed is presented comparatively in Table 2. Breast-conserving surgery (BCS) was performed more frequently in patients in group 1 (44.5%) than in groups 2 (13.2%) and 3 (11.9%) (*p* < 0.001). Mastectomy was performed mostly on patients with OMBD. The proportion of patients who underwent SLNB was 36.6% in group 1, 13.2% in group 2, and 9.2% in group 3 (*p* < 0.001). ALND was mostly performed on patients with oligo bone metastasis (group 2) and SLNB compared with patients without metastasis (group 1).

**Table 2:** Surgical treatment methods.

		Group 1	Group 2	Group 3	*p*
**Type of breast surgery**					
None	n (%)	8 (0.9)	6 (8.8)	36 (19.5)	< 0.001
Mastectomy		507 (54.6)	53 (77.9)	127 (68.6)	
Breast-conserving surgery		413 (44.5)	9 (13.2)	22 (11.9)	
**Axillary surgery**					
None	n (%)	16 (1.7)	8 (11.8)	39 (21.2)	< 0.001
ALND		433 (46.7)	49 (72.1)	112 (60.9)	
SLNB		339 (36.6)	9 (13.2)	17 (9.2)	
SLNB + ALND		139 (15.0)	2 (2.9)	16 (8.7)	
**SLNB method**					
Isosulfan Blue	n (%)	67 (14.2)	4 (30.8)	6 (17.1)	0.487
Radiocolloid		93 (19.7)	3 (23.1)	6 (17.1)	
Combined		312 (66.1)	6 (46.2)	23 (65.7)	
Tumor size (cm)	Median (range)	2.2 (0–16)	3 (0.7–16)	3 (0–14)	< 0.001
No. of positive SLNs	Median (range)	0 (0–11)	0 (0–6)	1 (0–7)	0.049
Number of SLNs removed	Median (range)	4 (0–12)	3 (0–8)	4 (1–16)	0.471
No. of lymph nodes removed by ALND	Median (range)	15 (1–71)	17 (1–53)	18 (0–57)	0.001
No. of positive nodes in ALND	Median (range)	0 (0–44)	6 (0–32)	4 (0–51)	< 0.001
**Perinodal involvement**					
No	n (%)	514 (79.0)	22 (47.8)	52 (57.8)	< 0.001
Yes		137 (21.0)	24 (52.2)	38 (42.2)	

*ALND* axillary lymph node dissection, *SLNB* sentinel lymph node dissection.

After ALND, the number of metastatic lymph nodes was 0 (0–44) in group 1, 6.0 (0–32) in group 2, and 4 (0–51) in group 3 (*p* < 0.001).

The median tumor size was 2.2 cm (range 0–16) cm in group 1, and 3.0 cm in group 2 (range 0.7–16 cm) and group 3 (range 0–14 cm) (*p* < 0.001).

The protocol and efficacy of adjuvant and neoadjuvant treatment on the groups are shown in Table 3.

**Table 3:** Adjuvant and neoadjuvant treatment of the groups.

Systemic therapies		Group 1	Group 2	Group 3	*p*
**Neoadjuvant treatment (NAT)**					
No	n (%)	822 (88.6)	61 (89.7)	147 (79.5)	0.003
Yes		106 (11.4)	7 (10.3)	38 (20.5)	
**Response to NAT**					
No	n (%)	8 (8.8)	4 (66.7)	6 (17.6)	NA
Partial		54 (59.3)	2 (33.3)	27 (79.4)	
Almost complete		14 (15.4)	0 (0)	1 (2.9)	
Complete		15 (16.5)	0 (0)	0 (0)	
**Adjuvant CT**					
No	n (%)	225 (29.6)	16 (27.6)	61 (38.9)	NA
Taxane and/or AC		517 (67.9)	41 (70.7)	92 (58.6)	
CMF		8 (1.1)	0 (0)	1 (0.6)	
Other		11 (1.4)	1 (1.7)	3 (1.9)	
**GCSF use**					
No	n (%)	285 (62.9)	29 (70.7)	54 (62.1)	0.588
Yes		168 (37.1)	12 (29.3)	33 (37.9)	
**Radiotherapy**					
No	n (%)	193 (22.6)	16 (26.7)	43 (30.9)	0.088
Yes		662 (77.4)	44 (73.3)	96 (69.1)	
**Hormonotherapy**					
No	n (%)	175 (20.2)	21 (31.8)	72 (44.2)	< 0.001
Tmx		248 (28.7)	17 (25.8)	45 (27.6)	
Aromatase Inh		398 (46.0)	25 (37.9)	40 (24.5)	
Switch		44 (5.1)	3 (4.5)	6 (3.7)	

*NA* not available, *CT* chemotherapy.

Neoadjuvant chemotherapy (NACT) was mostly performed on patients in group 3 (*p* = 0.003). The percentages of patients who received hormonotherapy after surgery were 79.8% in group 1, 68.2% in group 2, and 55.8% in group 3 (*p* < 0.001).

The histopathologic features of the tumors are compared in Table 4.

**Table 4:** The histopathologic features of the tumors.

Histopathologic features		Group 1	Group 2	Group 3	*p*
		n (%)	n (%)	n %	
**No. of tumor**					
Single	n (%)	776 (89.7)	48 (82.8)	137 (81.5)	0.019
Multiple		85 (9.8)	9 (15.5)	30 (17.9)	
Inflammatory		4 (0.5)	1 (1.7)	1 (0.6)	
**Carcinoma In situ**					
Yes	n (%)	346 (46.6)	23 (44.2)	42 (38.5)	0.285
No		397 (53.4)	29 (55.8)	67 (61.5)	
**Histology**					
IDC	n (%)	722 (77.8)	43 (63.2)	151 (81.6)	< 0.001
ILC		73 (7.9)	10 (14.7)	13 (7.0)	
Mixed		50 (5.4)	12 (17.6)	10 (5.4)	
Other		83 (8.9)	3 (4.4)	11 (5.9)	
**Histologic grade**					
1	n (%)	78 (10.6)	2 (4.1)	2 (1.6)	0.004
2		451 (61.0)	31 (63.3)	76 (59.4)	
3		210 (28.4)	16 (32.7)	50 (39.1)	
**Nuclear grade**					
1	n (%)	37 (6.0)	2 (5.3)	2 (2.2)	0.274
2		420 (67.7)	23 (60.5)	56 (62.9)	
3		163 (26.3)	13 (34.2)	31 (34.8)	
**Mitosis**					
1	n (%)	153 (25.5)	14 (37.8)	15 (17.0)	0.006
2		370 (61.8)	17 (45.9)	51 (58.0)	
3		76 (12.7)	6 (16.2)	22 (25.0)	
**ER**					
Neg	n (%)	243 (27.0)	20 (30.3)	74 (41.3)	0.002
1+		135 (15.0)	8 (12.1)	28 (15.6)	
2++		168 (18.7)	16 (24.2)	32 (17.9)	
3+++		354 (39.3)	22 (33.3)	45 (25.1)	
Percentage of ER	Median (Min–Max)	80 (1–100)	70 (5–100)	70 (2–100)	0.139
**PR**					
Neg	n (%)	271 (30.3)	23 (34.3)	65 (37.1)	0.03
1+		158 (17.7)	15 (22.4)	43 (24.6)	
2++		153 (17.1)	13 (19.4)	30 (17.1)	
3+++		312 (34.9)	16 (23.9)	37 (21.1)	
Percentage of PR	Median (Min–Max)	60 (0–100)	50 (0–100)	50 (1–100)	0.003
**cerbB2**					
Negative	n (%)	569 (77.4)	40 (75.5)	94 (67.6)	0.047
Positive		166 (22.6)	13 (24.5)	45 (32.4)	
**P53**					
Negative	n (%)	312 (40.1)	27 (44.3)	74 (50.7)	0.053
Positive		467 (59.9)	34 (55.7)	72 (49.3)	
**Ki67**					
≤ 14%	n (%)	439 (58.1)	26 (49.1)	61 (44.9)	0.01
> 14%		316 (41.9)	27 (50.9)	75 (55.1)	
Percentage of Ki67	Median (Min–Max)	15 (1–90)	15 (1–80)	25 (1–90)	< 0.001
**E-cadherin**					
Negative	n (%)	38 (9.9)	3 (13.0)	5 (8.5)	0.823
Positive		344 (90.1)	20 (87.0)	54 (91.5)	
**Lymph vessel invasion**					
No	n (%)	515 (76.9)	19 (42.2)	47 (48.0)	< 0.001
Yes		155 (23.1)	26 (57.8)	51 (52.0)	
**Blood vessel invasion**					
No	n (%)	558 (83.3)	32 (69.6)	62 (67.4)	< 0.001
Yes		112 (16.7)	14 (30.4)	30 (32.6)	
**Molecular classification**					
Luminal A	n (%)	319 (37.2)	20 (31.3)	36 (21.4)	0.003
Luminal B		364 (42.4)	30 (46.9)	79 (47.0)	
Triple negative		112 (13.1)	10 (15.6)	33 (19.6)	
HER2 enriched		63 (7.3)	4 (6.3)	20 (11.9)	

*IDC* invasive ductal carcinoma, *ILC* invasive lobular carcinoma, *ER* estrogen receptor, *PR* progestrone receptor.

The percentage of mixed-type tumor histology was 5.4% in group 1 and 3, and 17.6% in group 2. Invasive lobar carcinoma (ILC) and mixed-type tumors were more common in patients with OMBD (*p* < 0.001).

ER-positivity was ≥ 70% in groups 1 and 2 but progressively decreased below 60% in group 3 (*p* = 0.002). The percentage of PR positivity was highest in group 1 (60%) (*p* = 0.003). The median value of Ki67 was 25% in group 3 and was significantly higher compared with the other groups (*p* < 0.001). The rate of lymphoid and blood vessel invasion was similar in groups 2 and 3, and was significantly higher compared with group 1 (*p* < 0.001).

In the molecular classification, the luminal A subtype was most common in patients without metastasis (*p* = 0.003) with a rate of 37.2%, whereas the incidence of the luminal-B subtype was similar in all three groups.

The TNM staging was statistically different between the groups (*p* < 0.001) (Table 5). T1-T2 tumors and N0-N1 lymph nodes were most common in patients without metastasis (group 1), and T3-T4 tumors were most common in patients with OMBD (group 2).

**Table 5:** Comparison of cancer stages according to TNM classificatıon.

Stage	Group 1	Group 2	Group 3	*p*
	n (%)	n (%)	n %	
**T (TNM)**				
T1	395 (45.7)	11 (19.0)	35 (25.5)	< 0.001
T2	402 (46.5)	21 (36.2)	65 (47.4)	
T3	41 (4.7)	13 (22.4)	14 (10.2)	
T4	27 (3.1)	13 (22.4)	23 (16.8)	
**N (TNM)**				
N0	462 (51.5)	12 (20.3)	37 (25.9)	< 0.001
N1	262 (29.2)	13 (22.0)	32 (22.4)	
N2	109 (12.2)	14 (23.7)	39 (27.3)	
N3	64 (7.1)	20 (33.9)	35 (24.5)	
**Stage (TNM)**				
Stage 1	259 (30.5)	7 (11.1)	12 (7.3)	< 0.001
Stage 2	401 (47.2)	12 (19.0)	38 (23.2)	
Stage 3	189 (22.3)	29 (46.0)	62 (37.8)	
Stage 4	0 (0.0)	15 (23.9)	52 (31.7)	

All demographic, treatment-specific, histopathologic, and molecular variables that had statistical significance in univariate analysis were re-evaluated in multivariate logistic regression analysis.

The parameters that were statistically significantly different between the patients in groups 1 and 3 were evaluated in multiple regression analysis (Table 6). Those with a negative impact on patients in group 1 were as follows:

**Table 6:** Multivariate logistic regression analysis of demographic, therapeutic, and histopathologic parameters between Groups 1 and 3.

Parameter	B	Sig	Exp(B)	95% C.I. for EXP(B)
CEA *(continuous)*	0.052	0.006	1.053	1.015–1.093
Tumor size (cm) *(continuous)*	0.155	0.009	1.168	1.040–1.311
No NACT ***(ref.)***		0.134	1	
No response to NACT	22.897	0.999	8,788,932,664.350	0.000–
Partial response to NACT	0.842	0.021	2.320	1.137–4.734
Complete response to NACT	− 0.396	0.707	0.673	0.086–5.296
ER-negative ***(ref.)***		0.028	1	
ER(+)	− 0.632	0.086	0.531	0.258–1.094
ER(++)	− 0.490	0.154	0.613	0.312–1.202
ER(+++)	− 0.841	0.004	0.431	0.245–0.759
N0 ***(ref.)***		< 0.001	1	
N1	0.200	0.521	1.221	0.663–2.249
N2	1.118	0.001	3.058	1.591–5.878
N3	1.291	< 0.001	3.635	1.790–7.379
No local recurrence ***(ref.)***		0.052	1	
Recurrence in the opposite breast	0.578	0.316	1.782	0.575–5.519
Locoregional recurrence	1.283	0.024	3.609	1.188–10.964
Constant	− 2.736	< 0.001	0.065	

*NACT* neoadjuvant chemotherapy, *N* nodal involvement.

Every 1 unit rise in CEA values and every 1 cm increase in tumor size enhanced the risk of multiple metastases by 1.05 and 1.17 times. These significant increases in risk were independent of neoadjuvant therapy, ER, N, and local recurrence variables in the model. Also, for group 1, the risk of multiple metastatic disease increased 2.3 times in patients with a partial response to neoadjuvant therapy, 3.1 and 3.64 times in patients with N2 and N3 nodal involvement, and 3.6 times in patients who developed loco-regional recurrence. By contrast, patients in group 1 with ER (+++) positive tumors were protected 0.43 times against the risk of multiple metastases.

The multivariate logistic regression analysis of demographic, therapeutic, and histopathologic parameters between groups 1 and 2 is shown in Table 7. For the patients in group 1, the risk of OMBD increased 7.7 and 5.4 times in patients with T3 and T4 tumors, and 2.7 times in those with perinodal invasion of the primary tumor. Also, every 1 unit rise in CEA values increased the risk of OMBD by 1.08 times. The most remarkable finding was the 68.3-fold increased risk of transition from a nonmetastatic state to OMBD in patients who developed locoregional recurrence.

**Table 7:** Multivariate logistic regression analysis of demographic, therapeutic and histopathological parameters between Group 1 & 2.

Parameter	B	Sig	Exp(B)	95% C.I.for EXP(B)
CEA *(continuous)*	0.077	0.014	1.081	1.016–1.149
No HT ***(ref.)***		0.161		
HT (Tamoxifen)	0.121	0.859	1.129	0.297–4.288
HT (Aromatase Inhitor)	0.164	0.791	1.179	0.350–3.965
HT (Switch)	2.224	0.035	9.248	1.164–73.475
Perinodal invasion *(ref.none)*	0.984	0.043	2.675	1.033–6.929
Lymphovascular invasion *(ref. none)*	0.768	0.113	2.156	0.834–5.571
T1 ***(ref.)***		0.010		
T2	0.334	0.557	1.396	0.458–4.258
T3	2.037	0.004	7.670	1.591–30.539
T4	1.678	0.046	5.357	1.033–27.778
No local recurrence ***(ref.)***		< 0.001		
Recurrence in the opposite breast	− 18.737	0.998	0.000	0.000–
Locoregional recurrence	4.224	< 0.001	68.292	10.441–446.667
Constant	− 4.627	< 0.001	0.010	

*HT* hormone therapy, *T* T category.

As a result, T3-T4 tumors, perinodal tumor invasion, and high CEA levels in patients without metastasis (group 1) were factors that triggered the development of OMBD. The risk of OMBD increased 68 times in patients who developed locoregional recurrence during follow-up.

OMBD was present at the time of primary breast cancer diagnosis in 18 patients (synchronous OMBD), and it occurred during follow-up in the other 50 patients (asynchronous OMBD).

In our series, we had 39 patients with SBM and 29 patients with more than one bone metastasis. When these two strata of the OMBD group were compared, with the analysis being limited to the total number of patients (n = 68), no significant difference was found between them in terms of demographic, treatment-specific, histopathologic, and molecular variables.

The survival outcomes were statistically significantly different between the groups (*p* < 0.001) (Table 8).

**Table 8:** Survival outcomes.

Overall survival	Group 1	Group 2	Group 3	*p*
	n (%)	n (%)	n (%)	
Deceased	136 (14.7)	51 (75.0)	141 (76.2)	< 0.001
Alive	792 (85.3)	17 (25.0)	44 (23.8)	

**Figure 2:**
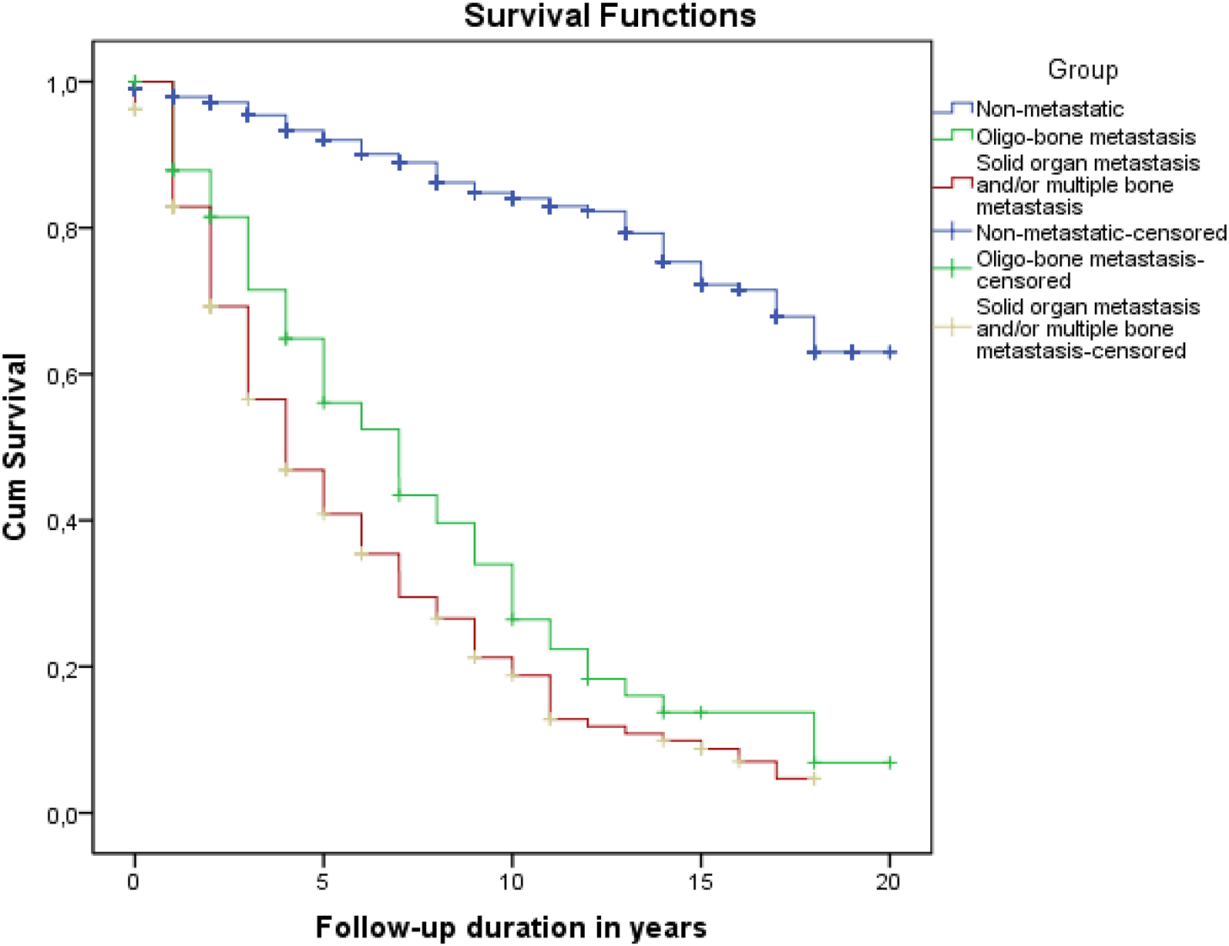
The overall survival of Groups 1, 2, and 3.

The mean and median follow-up for the entire study group was 7.0 ± 5.4 and 6.0 (range 0–20) years. The mean OS of the groups was 16.7 ± 0.3 years in group 1, and 7.8 ± 0.8 and 5.9 ± 0.4 years in groups 2 and 3, respectively (*p* < 0.001 for the comparison of all three groups together; *p* < 0.001 for group 1 vs. 2 and 3) and (*p* = 0.037 for group 2 vs. group 3) (Fig. 2).

**Figure 3:**
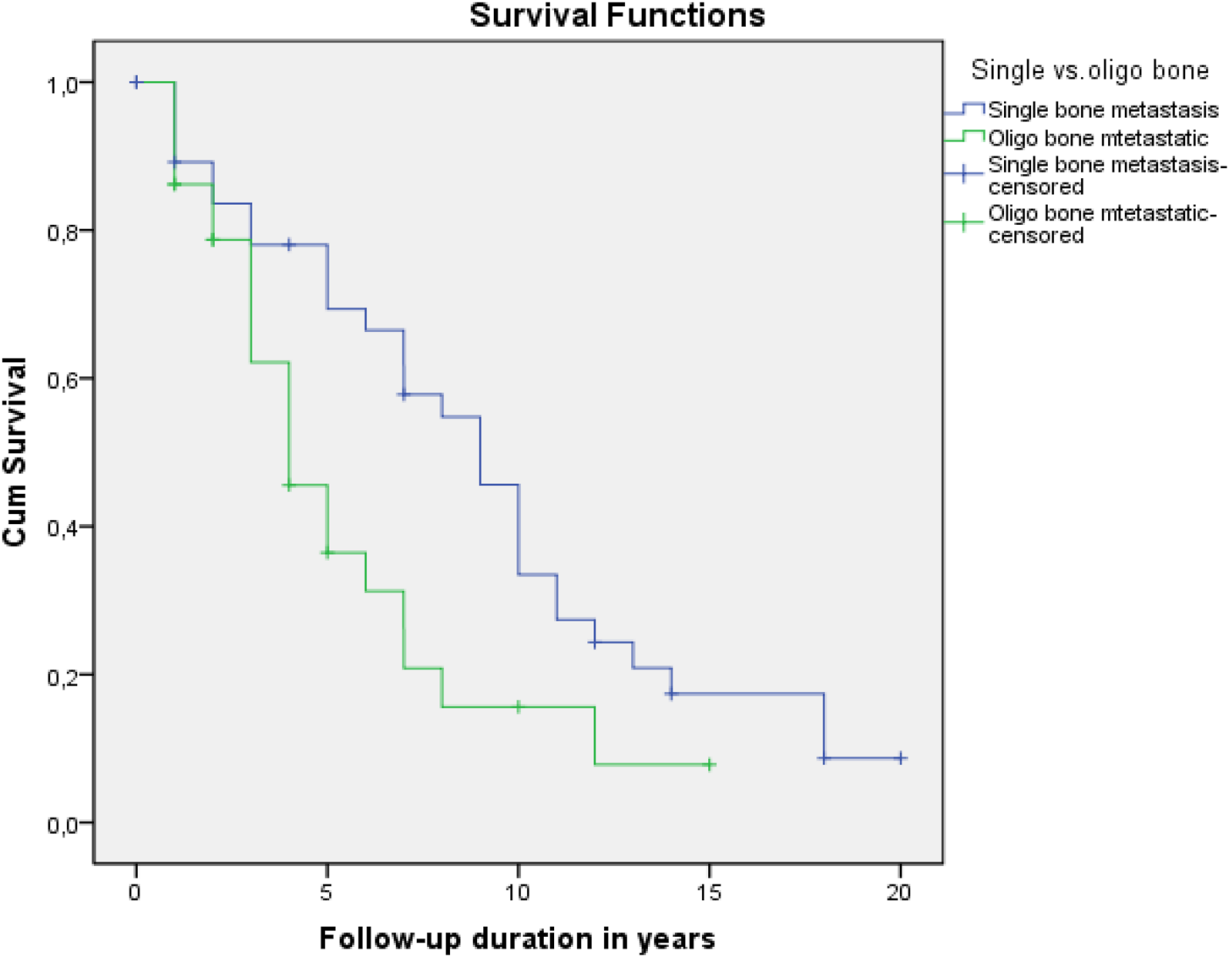
Survival outcomes of patients with SBM and > 1 bone metastasis.

In the subgroup survival analysis of patients in group 2 (OMBD), the mean and median survival was 5.5 ± 0.8 and 4.0 ± 0.8 years vs. 9.2 ± 0.98 and 9.0 ± 1.05 years in patients with more than one bone metastasis and SBM, respectively (*p* = 0.019) (Fig. 3).

**Figure 4:**
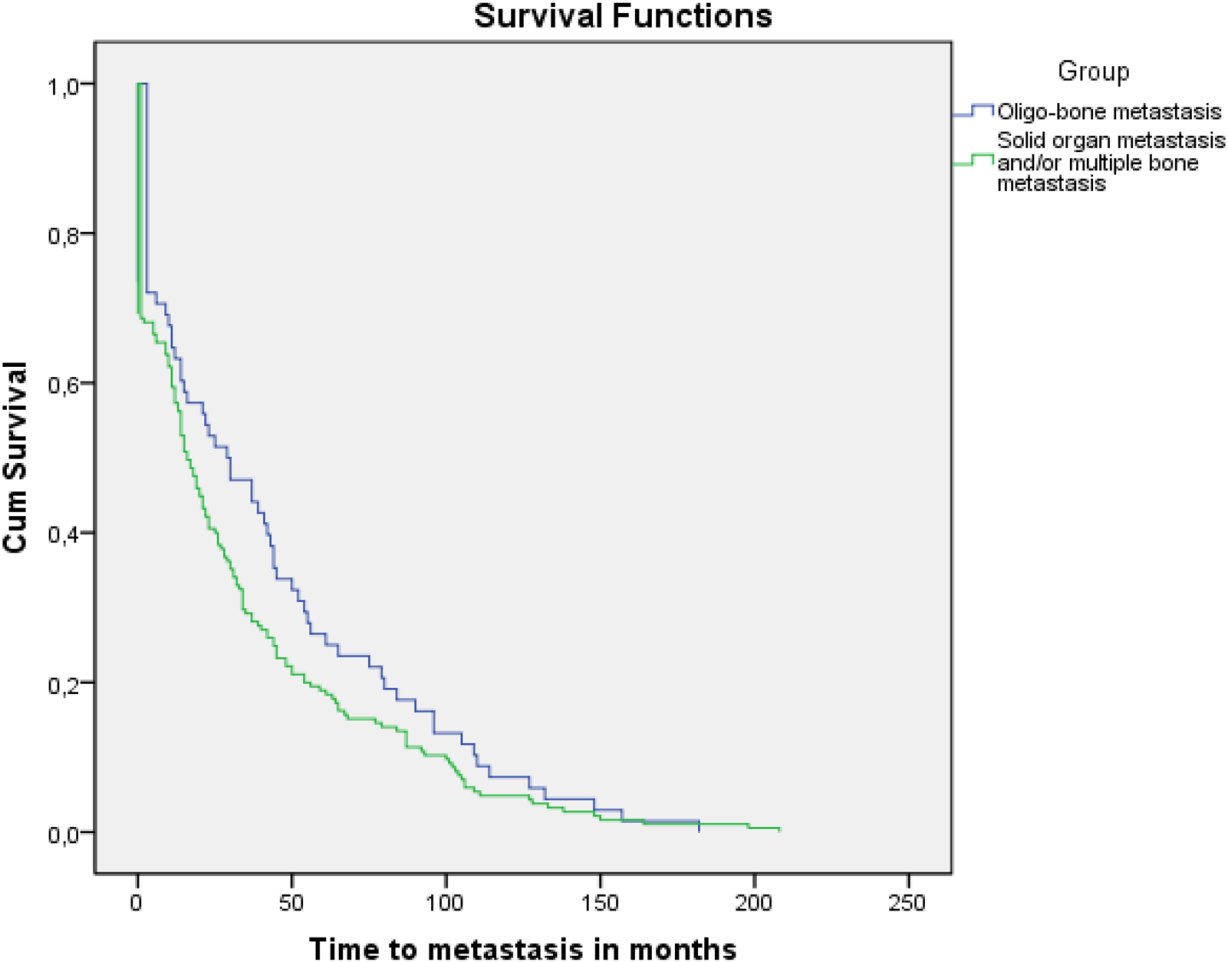
Time from primary diagnosis of breast cancer to the occurrence of OMBD or multimetastatic disease. (OMBD: 41.9 ± 5.5 months, widespread metastatic disease: 32 ± 3.0 months; *p* = 0.143).

**Figure 5:**
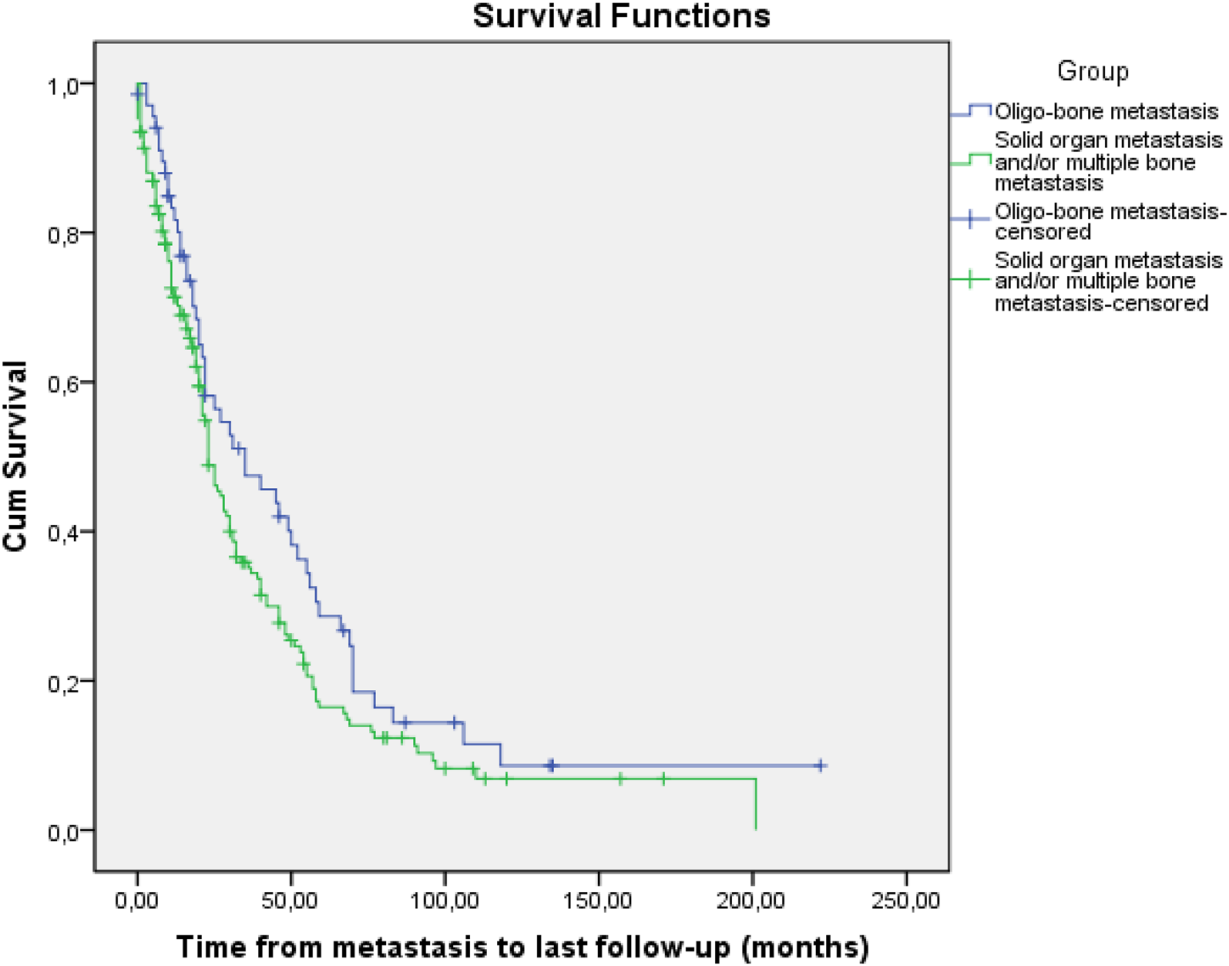
Overall survival of patients with OMBD and multimetastatic disease after the occurrence of metastasis. (OMBD: 54.4 ± 8.2 months, widespread metastatic disease: 41.3 ± 4.3 months; *p* = 0.073).

Here, we noted that survival times from breast cancer diagnosis differed depending on whether patients developed OMBD or other sites of metastasis. We also analyzed the model in which patients developed metastases for the first time, revealing whether there was a difference in the time of their occurrence. We then calculated the OS after the development of widespread metastases and metastases to bone. The time from the primary diagnosis of breast cancer to the occurrence of OMBD and multimetastatic disease (MMD) was 41.9 ± 5.5 months and 32.0 ± 3.0 months (*p* = 0.14), respectively (Fig. 4). The outcome analysis after the development of metastas is revealed a mean survival of 54.4 ± 8.2 versus 41.3 ± 4.3 months (*p* = 0.073) in OBMD and MMD groups (Fig. 5).

## Discussion

In our previous study, we analyzed demographic, epidemiologic, histopathologic, and intrinsic tumor subtype differences between 863 patients with breast cancer without metastasis and 47 patients with breast cancer with SBM ≥ 6 months after their first diagnosis. Among the established risk factors, we studied 29 variables and found that the risk of developing SBM was approximately 4.8 and 2.8 times higher in patients with breast cancer with TNM Stage III tumors and with mixed-type (invasive ductal carcinoma + ILC) histology^10^. Following this study, and again in our patient series, we aimed to evaluate patients without metastases and those with OMBD according to demographic, epidemiologic, histopathologic, and intrinsic tumor subtypes. Thus, we planned to identify the common characteristics of patients with SBM and OMBD and to reveal whether OMBD was a different entity or a more aggressive form originating from isolated bone disease (SBM). Although ILC and mixed-type tumors were found to be significantly higher in patients with OMBD (17.6% vs. 5.4%, *p* < 0.001) compared with other groups in univariate analysis, this feature lost its significance in multivariate regression analysis. In the present study, the most important risk factors for the development of OMBD in the non-metastatic patient group were as follows: T3–T4 tumor, perinodal tumor invasion, and particularly postoperative locoregional recurrence. When we compared these results with our previous study^10^, the common feature in our patients with SBM and OMBD was the development of both following advanced stage tumors (Stage IIIA and B).

In 1995, Hellman et al. first described oligometastasis and suggested that, at this stage, cancer had not yet reached its full metastatic potential and was restricted to certain regions^9^. In other words, the concept of oligometastatic disease implies that few metastases, usually under five, may be present before tumor cells reach diffuse metastatic potential^11^. In this context, patients with breast cancer with oligometastasis have so far been considered to have a disease with favorable course, which should be treated with curative intent^12^.

Herein, we examined the clinical course of patients with oligometastatic disease in our large series of patients and compared the results with the literature. Our definition of OMBD is the presence of solitary or fewer than five detectable lesions limited to a single organ amenable to local treatment with curative intent. Patients with breast cancer with bone-only metastasis have a fairly good prognosis with an average survival of 24–65 months after metastasis is detected^7,13,14^. In our study, the mean OS was 7.8 ± 0.8 years in the patient group with OMBD.

In our previous study on patients with SBM, the mean and median survival times were 9.9 and 7.0 years, respectively^10^. In the present study, the mean and median OS of patients with > 1 bone metastasis was 5.5 ± 0.8 and 4 years and was significantly shorter than in those with SBM (9.2 ± 0.98 and 9 years, respectively) (*p* = 0.019). This result indicates that patients with breast cancer with OMBD do not have similar outcome features and a favorable prognosis like those with SBM.

Parkes et al. reported a similar result. They evaluated 1445 patients with bone-only metastasis followed for at least 6 months at MD Anderson Cancer Center from 1997 to 2015 and reported poorer OS in patients with multiple bone metastases (median OS, 4.80 years; 95% CI 4.49–5.07) compared with SBM (median OS, 7.54 years; 95% CI 6.28–10.10)^15^. In addition, in a systematic review examining prognostic factors on survival in patients with oligometastatic breast cancer, solitary metastasis was associated with better OS^8^. In a study of 50 patients with extracranial oligometastatic breast cancer, those with single metastasis highly benefited from systemic chemotherapy and surgical resection and gained survival advantage with statistical significance^16^.

Our study has several limitations. First, it is a single institute series. Although 43 prognostic and confounding factors were analyzed in depth in our study, the small number of patients with OMBD prevented us from revealing the distinctive biologic characteristics of these patients. With our data, we were able to show that single bone metastatic disease and OMBD were not similar entities. However, we could not identify any molecular marker that would show whether a transition period existed between them.

## Conclusion

OMBD seems to be a different disease than breast cancer with isolated bone metastases. The high risk of developing OMBD, especially following locoregional recurrence, increases the importance of locoregional therapy in large T and N stage tumors.

Larger case groups are needed to clarify whether these two subgroups, including patients with SBM and OMBD, have different determinants.

## Data Availability

The data of all patients are kept by the corresponding author and are available through him.
